# Generation of an anti-desmoglein 3 antibody without pathogenic activity of pemphigus vulgaris for therapeutic application to squamous cell carcinoma

**DOI:** 10.1093/jb/mvy074

**Published:** 2018-09-18

**Authors:** Shin-Ichi Funahashi, Shigeto Kawai, Etsuko Fujii, Kenji Taniguchi, Kiyotaka Nakano, Shumpei Ishikawa, Hiroyuki Aburatani, Masami Suzuki

**Affiliations:** 1 Forerunner Pharma Research Co., Ltd., Komaba Open Laboratory, The University of Tokyo, 4-6-1 Komaba, Meguro-ku, Tokyo, Japan; 2 Chugai Pharmaceutical Co., Ltd., 200 Kajiwara, Kamakura, Kanagawa, Japan; 3 Genome Science, RCAST, The University of Tokyo, 4-6-1 Komaba, Meguro-ku, Tokyo, Japan

**Keywords:** DSG3, epitope, monoclonal antibody, pemphigus vulgaris, squamous cell carcinoma

## Abstract

It is ideal for the target antigen of a cytotoxic therapeutic antibody against cancer to be cancer-specific, but such antigens are rare. Thus an alternative strategy for target selection is necessary. Desmoglein 3 (DSG3) is highly expressed in lung squamous cell carcinoma, while it is well-known that anti-DSG3 antibodies cause pemphigus vulgaris, an autoimmune disease. We evaluated DSG3 as a novel target by selecting an epitope that exerts efficacy against cancer with no pathogenic effects in normal tissues. Pathogenic anti-DSG3 antibodies induce skin blisters by inhibiting the cell–cell interaction in a Ca^2+^-dependent manner. We screened anti-DSG3 antibodies that bind DGS3 independent of Ca^2+^ and have high antibody-dependent cell cytotoxicity (ADCC) activity against DSG3-expressing cells. These selected antibodies did not inhibit cell–cell interaction and showed ADCC activity against squamous cell carcinoma cell lines. Furthermore, one of the DSG3 antibodies showed anti-tumour activity in tumour mouse models but did not induce adverse effects such as blister formation in the skin. Thus it was possible to generate an antibody against DSG3 by using an appropriate epitope that retained efficacy with no pathogenicity. This approach of epitope selection may expand the variety of druggable target molecules.

Therapeutic antibodies are being actively researched and used to treat an increasing number of diseases, including cancer (1). Therapeutic antibodies for cancer are now available with neutralizing, antibody-dependent cellular cytotoxicity (ADCC) and complement-dependent cytotoxicity (CDC) functions (2–6). Technologies for producing low-fucosylated or defucosylated antibodies that have enhanced ADCC have been established and applied to therapeutic antibodies (7–9). The ideal target molecule for a cytotoxic antibody is a cancer-specific antigen, but although research has focused on searching for such a target, almost no cancer-specific antigens have been discovered (10). Thus, it may be necessary to discover novel therapeutic antibodies by an alternative strategy that involves selecting an antigen with relatively high expression in cancer compared with normal tissue and designing an antibody with the appropriate anti-cancer functions against that antigen.

Desmoglein-3 (DSG3) is expressed in normal squamous epithelia and overexpressed in squamous cell carcinoma (SCC) of the lung (11–14). However, DSG3 is a molecule associated with pemphigus vulgaris (PV), a severe autoimmune blistering disease affecting skin and mucosa, and it is known that anti-DSG3 autoantibodies are involved in the onset of the disease (15, 16). Different disease states are induced according to the different recognition sites of the anti-DSG3 antibodies. Evidence of this is that the most severe disease state is induced by antibodies that recognize the interaction sites of the EC1-EC2 domains at the N-terminal of DGS3, whereas antibodies with other recognition sites are not associated with pathogenic states (17–19). Because the evidence from PV suggests that there is a large difference in biological reaction according to the recognition site of an autoantibody, we hypothesized that selecting the antibody epitope carefully would make it possible to develop a novel anticancer antibody applicable for SCC that avoids the pemphigus pathogenesis.

Here we show that we have obtained an anti-mouse DSG3 monoclonal antibody (mAb) with therapeutic potential and have evaluated whether its pathogenic activity had been successfully separated from its anticancer activity. Furthermore we have attempted to generate an anti-human DSG3 mAb with the same characteristics.

## Materials and Methods

### Human clinical samples

The tissues evaluated in the current study are from the tissue library at PharmaLogicals Research Pte. Ltd. (PLR, Singapore) (20). The tissues examined in the current study included six cases of SCC (lung 2, skin 3 and uterus 1) and six cases of lung adenocarcinoma. The surgically excised tissues were provided by patients that gave their informed consent, as approved by the ethical committee at PLR in Singapore.

### Animals

Dsg3 (-/-) mice (B6; 129X1-*Dsg3^tm1Stan^*/J) were purchased from Jackson Laboratory and bred at Charles River Laboratories Japan. Severe combined immunodeficient (SCID) mice (C.B-17/lcr-*scid* Jcl) were purchased from CLEA Japan. MRL/lpr mice (MRL/MpJ-*Tnfrsf6*^lpr^/Crlj) and Balb/c mice (BALB/cAnNCrlCrlj) were purchased from Charles River Laboratories Japan. Care and use of animals used in this study was in accordance with the Guidelines for the Care and Use of Laboratory Animals under the approval of the Institutional Animal Care and Use Committees at The University of Tokyo and Chugai Pharmaceutical Co., Ltd. Immunizations were performed in the experimental animal facility at The University of Tokyo. Mouse xenograft experiments were performed at Chugai Pharmaceutical Co., Ltd.

### Cells

Chinese hamster ovary (CHO) cell line DG44, mouse B cell line Ba/F3 or human lung SCC cell lines HARA and A431 were purchased from Invitrogen, Riken Bioresource Center, and Health Science Research Resources Bank, respectively. Human NK cell lymphoma cell line NK-92, tongue SCC cell lines SCC-15 and mouse myeloma P3-X63Ag8U1 (P3U1) were obtained from the ATCC. Mouse lung SCC cell line LC-12 was purchased from NCI and maintained by subcutaneously inoculating minced tissues into Balb/c mice. Normal human keratinocyte CryoNHEK-Neo and mouse keratinocyte MPEK-BL6 were purchased from Lonza and CELLnTEC. GDP-fucose transporter (GFT) (-/-) CHO cell was obtained from Chugai Pharmaceutical Co., Ltd. (7). All cell cultures were conducted according to the manufacturers’ instructions. Most of the reagents for cell culture were purchased from Invitrogen. Culture conditions are described in Supplementary Data.

### DNA

Full-length cDNA of human DSG3 (RefSeq: NM_001944) was obtained by PCR amplification from human small intestine Marathon-Ready cDNA (Clontech). In the same manner, mouse DSG3 cDNA (RefSeq: NM_030596) was cloned from mouse E17 cDNA (Clontech). Mouse FcγRIIIa (CD16) (RefSeq: NM_010188) was cloned from mouse spleen cDNA (Clontech).

Each cDNA was cloned into the mammalian expression vector, pMCN (pMCN/human DSG3, pMCN/mouse DSG3). pMCN is vector driven under the promoter of mouse CMV and is selected with the neomycin-resistance gene.

### Establishment of DSG3-expressing stable cell lines in CHO and Ba/F3 and NK92 cell lines for the ADCC assay

All transfections were carried out by electroporation with BioRad GenePulser, and stable transfectants were selected in the medium in the presence of geneticin (Invitrogen). Next, cell lines derived from single cells that had been clonally expanded were obtained by the limiting-dilution method. Electroporation was conducted under conditions of 1.5 kV, 25 μF for CHO DG44, 260 V, 1050 μF for Ba/F3 and 200 kV, 975 μF for NK92. The cells were suspended in phosphate-buffered saline (PBS) (-) at a concentration of 1 × 10^7^ cells/ml, and were transfected with 10–25 μg of plasmid DNA. The cell surface expression of mouse or human DSG3 in the transfected cells was confirmed by flow cytometry (FACS) with AK23 antibody. CHO DG44 transduced with mouse or human DSG3 cDNA was designated as mouse DSG3/DG44 or human DSG3/DG44, respectively. The Ba/F3 transfectants were designated as mouse DSG3/BaF3 and human DSG3/BaF3.

To establish a stable assay for ADCC activity, NK92 expressing mouse FcγRIIIa (mFcγRIIIa-NK92) was established as a cell line and incorporated as effector cells. Mouse FcγRIIIa was expressed as a chimeric protein comprising the extracellular domain of mouse FcγRIIIa (aa 1-212) fused to the transmembrane domain and the cytoplasmic domain of human FcγRIIIa (aa 207-254). The cells were clonally expanded by selecting in 500 μg/ml of geneticin.

### Generation of recombinant DSG3 proteins

Soluble mouse DSG3-His proteins were expressed in DG44 and affinity purified from the supernatants of the transfectants with His-Trap column (GE Healthcare).

Soluble human DSG3 was expressed as sDSG3-mIgG2aFc composed of the extracellular domain of human DSG3 (aa 1-616) and the Fc portion of mouse IgG2a, and was purified with HiTrap Protein G HP column (GE Healthcare) and eluted with 0.1 M Glycine-HCl (pH 2.7). The eluate was gel-filtrated with Superdex 200HR 10/30 (GE Healthcare).

Glutathione S-transferase (GST)-human DSG3 fusion protein (GST-hDSG3) was expressed in *Escherichia coli* as a fusion of GST and 125 aa of human DSG3 (aa 491-615) with His-tag. GST-hDSG3 was purified with His-Trap column (GE Healthcare) for use as an antigen for ELISA.

### Immunohistochemical (IHC) analysis of human DSG3 in clinical samples

Human tissues were fixed in 4% paraformaldehyde upon collection, and embedded in paraffin by the AMeX method as described previously (21, 22). Thin sections were prepared at a thickness of 3–5 µm for histology and IHC. IHC staining for human DSG3 in human tissues was performed using the following method. A monoclonal mouse anti-human DSG3 antibody (Clone 5G11, Zymed) was applied as the primary antibody. The tissues were stained by an indirect immunoperoxidase method using the Ventana HX Discovery System (Ventana Medical Systems). Briefly, the slides were de-waxed and treated with protein block (Dako Cytomation) to reduce non-specific staining and 3% H_2_O_2_ in methanol to block endogenous peroxidase. After incubation with the primary antibody and Discovery Universal Secondary Antibody (Ventana Medical Systems), streptavidin conjugated to horseradish peroxidase (Ventana Medical Systems) was applied and the reaction visualized with a diaminobenzidine solution (Ventana Medical Systems). The slides were counterstained with haematoxylin and coverslipped. The slides were read under a light microscope.

The slides were read for staining frequency (positive percentage to all tumour cells), and staining intensity (scores: 0, negative; 1, very weak; 2, weak; 3, moderate; 4, strong). The staining score was calculated by adding up the product of staining frequency to intensity scores.

### Generation of anti-mouse DSG3 mAbs

mAbs against mouse DSG3 were generated by DNA immunization. A plasmid DNA expressing full length mouse *Dsg3* was inoculated into the skin of the abdomen of *Dsg3*-knockout (KO) mice using Helios Gene Gun (BioRad) followed by intravenous injection of mouse DSG3/DG44 cells as a booster immunization. At 4 days after the last immunization, splenocytes were isolated and fused with mouse myeloma P3U1 by the general polyethylene glycol method with PEG1500 (Roche Diagnostics) (23). The resulting hybridomas were selected by culturing in hypoxanthine/aminopterin/thymidine (HAT) medium containing RPMI-1640 medium with 10% FBS, 1-fold concentration of HAT media supplement (Sigma) and 0.5-fold concentration of BM-combined H1 Hybridoma cloning supplement (Roche Diagnostics). The culture supernatants were screened for their ability to bind to mouse DSG3 by FACS with mouse DSG3/DG44. Positive hybridomas were subcloned by the limited-dilution method. We selected 34 FACS-positive clones, measured their ADCC activity and further selected 12 candidates with strong ADCC. The 12 hybridomas, 1-2, 18-1, 33-1, 32-2, 29-2, 10-2, 20-1, 37-1, 5-1, 39-2, 19-1 and 40-2 were cultured in the medium containing HAT medium supplemented with Ultra low IgG FBS (Invitrogen), and mAbs were purified by HiTrap Protein G column (GE Healthcare). Isotyping was carried out with IsoStrip Mouse Monoclonal Antibody Isotyping Kit (Roche Diagnostics).

### Generation of anti-human DSG3 mAbs

Anti-human DSG3 mAbs were generated by immunizing MRL/lpr mice and Balb/c mice of 7–8 weeks old. At the first immunization, 100 μg of soluble human DSG3 (sDSG3-mIgG2aFc) was mixed with complete Freund’s adjuvant (Beckton Dickinson) and was inoculated subcutaneously. Two weeks later, 50 μg of sDSG3-mIgG2aFc was mixed with incomplete Freund’s adjuvant and was inoculated subcutaneously. At 1 week intervals, booster immunization was performed two to four times, and a final immunization was carried out by injecting 50 μg of the same protein into the tail vein. At 4 days after final administration, splenocytes were isolated and fused with P3U1 by the conventional method. The hybridoma supernatant was screened for binding activity to human DSG3 by FACS with human DSG3/DG44. Positive hybridomas were cloned by the limited-dilution method to establish monoclonal antibodies specific for human DSG3. Of the antibody clones, DF366 was selected as the mAb with the strongest ADCC activity.

### FACS

Binding affinity of mAbs to the antigens was analysed as described previously (24, 25). Briefly, Ba/F3 or DG44 transfectants were incubated with mAb diluted to appropriate concentrations for 1 h on ice, and then with FITC-labelled anti-mouse IgG for 30 min on ice. After reaction, the cells were analysed by FACS on a FACSCalibur flow cytometer (Beckton Dickinson).

In general, antibodies that induce PV-like states are known to show Ca^2+^-dependent binding to mouse DSG3. Thus to select antibodies with no pathogenic activity, we screened mAbs by evaluating their Ca^2^^+^ independent binding activity. The mAbs were analysed as with 1 mM or without Ca^2+^ and 5 mM EDTA by FACS.

In the case of LC-12 cells, the tumour tissue was resected and disaggregated into a single cell suspension by treatment with Dispase II (Roche Diagnostics) at 37°C for 20 h.

### ELISA

For GST-DSG3 ELISA, GST-hDSG3 protein was coated onto the microtiter plate at a concentration of 1 μg/ml, and blocked with 1% BSA. Then anti-human DSG3 antibodies were incubated with GST-hDSG3. The bound antibodies were detected with alkaline phosphatase (AP)-conjugated goat anti-mouse kappa antibody (Southern Biotech).

In order to classify the antibodies to subgroups by binding regions the competitive ELISA was performed as follows. After preincubation with non-labelled antibody (test antibody), biotin-labelled antibody was added, then the binding inhibition by test antibody was measured to classify the binding region. Anti-human DSG3 mAbs DF029, DF129, DF131, DF132, DF138, DF187 and DF269 were biotinylated. Biotinylation was conducted with a biotin labelling kit (Roche Diagnostics). The reaction was performed as reported before (26). Briefly, sDSG3-mIgG2aFc were bound onto anti-mouse IgG2a antibody-precoated microtiter plate. Then test antibodies were incubated at room temperature for 1 h, followed by incubation with biotinylated antibodies. After washing, the binding of biotinylated antibodies was detected with AP-conjugated streptavidin (Zymed). The mAbs with 90% of binding inhibition were classified as antibodies with the same binding region.

### Generation of ADCC-enhanced antibody

Mouse mAbs were genetically engineered to produce antibodies of the IgG2a isotype. Total RNA was extracted from the hybridoma with the RNA easy plant mini kit (QIAGEN), and the genes encoding the mAb were amplified by reverse transcription-PCR (RT-PCR) using the method described previously (26). PCR products were cloned into pGEM-T Easy vector (Promega) and nucleotide sequences were determined.

A chimeric anti-DSG3 antibody, in which the mouse variable regions were linked with the mouse IgG2a and kappa constant regions or human IgG1 and kappa regions, was constructed. The light and heavy chain expression vectors were co-transfected into CHO DG44 cells and selected with geneticin. Screening clones of the well-expressed antibodies by flow cytometry with mouse DSG3/DG44 or human DSG3/DG44, we purified the chimeric antibodies with a HiTrap ProteinG column. Anti-mouse DSG3 mAb 18-1 with a mouse IgG2a isotype was designated as 18-1m, and anti-human DSG3 mAb DF366 as DF366m. Anti-human DSG3 mAb DF366 with a human IgG1 isotype was designated as DF366c.

Defucosylated antibody 18-1m or DF366m was expressed in GFT (-/-) CHO cells with disrupted GFT alleles of GFT gene (7) and designated as df-18-1m or df-DF366m.

### Analysis of cell-cell dissociating activity

Cell–cell dissociating activity of mAbs was analysed by the keratinocyte dissociation assay described previously (27). Normal mouse keratinocytes (MPEK-BL6) or human keratinocytes (CryoNHEK-Neo) were used. When cells reached confluence, the culture medium was changed to the same medium containing 1.2 mM Ca^2+^. Then antibody was added to a final concentration of 10 μg/ml. After incubation at 37°C overnight, Staphylococcal exotoxin, exfoliative toxin A (ETA) (Toxin Technology) was added at 0.5 ug/ml for over 2 h to disrupt DSG1. Cells were washed with HBSS twice, and incubated with Dispase (Roche Diagnostics) for 30 min to detach the monolayer sheets. The detached cells were further mechanically dissociated by pipetting. After pipetting, the cells were observed by light microscopy.

AK23 antibody, which was derived from an active PV mouse model (27) and is known to inhibit cell–cell interaction, was used as the positive control for this assay.

Mouse IgG2a mAb AK23m was generated by a similar process to 18-1m, using a published sequence of the variable region of mAb AK23, which induces PV (19, 28).

### Determination of ADCC

ADCC activity of mAbs was determined as follows. Tumour cells were cultured in RPMI1640 with penicillin/streptomycin and 10% FBS (RPMI medium). About 1 × 10^6^ cell of mouse DSG3/BaF3 or human DSG3/BaF3 was suspended in 200 μl of RPMI medium with 3.7 MBq of ^51^Cr-sodium chromate (GE Healthcare) and incubated in 5% CO_2_, at 37°C for 1 h. Cells were washed with RPMI medium adjusted to a concentration of 2 × 10^5^ cells/ml and dispensed at 50 μl into a 96-well U-bottomed plate. Then 50 μl of antibody solution was added to each well, and the plates were incubated for 15 min at room temperature. Next 5 × 10^4^ cells of mFcγRIIIa-NK92 (in the case of mouse IgG2a chimeric antibodies) or NK92 (in the case of human IgG1 chimeric antibodies) were added as effector cells. The tumour cells were further incubated at 37°C for 4 h in 5% CO_2_. 100 μl of supernatants were collected from each well and radioactivity in the supernatants was measured with a gamma counter (1480 WIZARD 3″, Wallac). ^51^Cr release was calculated based on the following formula:

$${\text{Specific}\;}^{51}\text{Cr release}\;\left(\%\right)=(A-C)/(B-C)\;\times \;1\mathrm{00}$$

where *A* is the ^51^Cr release of each well (cpm), *B* is the mean ^51^Cr release for 50 μl of cells incubated in 150 μl of 2% Nonidet P-40 (Nakalai Tesque) and *C* is the mean ^51^Cr release for 50 μl of cells incubated in 150 μl of RPMI medium (cpm). All experiments were conducted in duplicate. Median value and standard deviation were calculated. Mouse IgG2a (Becton Dickinson) was used as a negative control antibody.

### *Determination of anti-tumour efficacy and toxicity* in vivo

The anti-tumour activity of anti-mouse DGS3 antibodies was evaluated using a syngeneic mouse model as follows. Balb/c mice were inoculated subcutaneously with approximate 3 mm^3^ cubes of LC-12 tumour tissue. Mice were divided into three groups one day after tumour inoculation. Each group consisted of 10 mice, and 18-1m, df-18-1m (10 mg/kg) or vehicle (PBS) was administered intravenously on days 1, 8 and 15. Tumour volume and body weight were measured twice a week.

Anti-tumour efficacy and toxicity were evaluated in human SCC xenograft models. About 1 × 10^7^ cells of HARA and A431 cells suspended in HBSS were inoculated and approximate 3 mm^3^ cubes of SCC-15 tumour tissue were inoculated subcutaneously into SCID mice. When the mean tumour volumes reached 100 mm^3^, 80 mm^3^ and 120 mm^3^, respectively, the mice were subjected to the study. Seven and five SCID mice per group (HARA and A431) were dosed intravenously with 10 mg/kg of mAb (df-DF366m) or with vehicle (PBS) once a week. Four SCID mice per group (SCC-15) were dosed intraperitoneally with 10 mg/kg of mAb (df-DF366m) or vehicle (PBS) once a week for 5 weeks. Tumour volume and body weight were measured twice a week. Tumour volume was determined with the formula: *ab^2^*/2 (mm^3^), where *a* and *b* are the longest and shortest diameters, respectively. Statistical analysis was conducted with the Dunnett’s test (LC-12) and *t*-test (HARA, A431 and SCC-15) using the SAS statistical package software (SAS Institute). Statistical significance was judged at *P* < 0.05.

At necropsy, the animals were euthanized by exsanguination from the abdominal artery under deep isoflurane anaesthesia. The small intestine, colon, liver, kidney, spleen and thymus, and tissues with squamous epithelia (skin, oral mucosa, oesophagus and forestomach) were fixed in 10% neutral-buffered formalin, and embedded into paraffin by a routine method. Thin sections were prepared at a thickness of 5 µm, stained with haematoxylin and eosin, and read under a light microscope. Inflammatory cell infiltration was evaluated pathologically by certified pathologists.

## Results

### DSG3 expression in human tissues

**Fig. 1:**
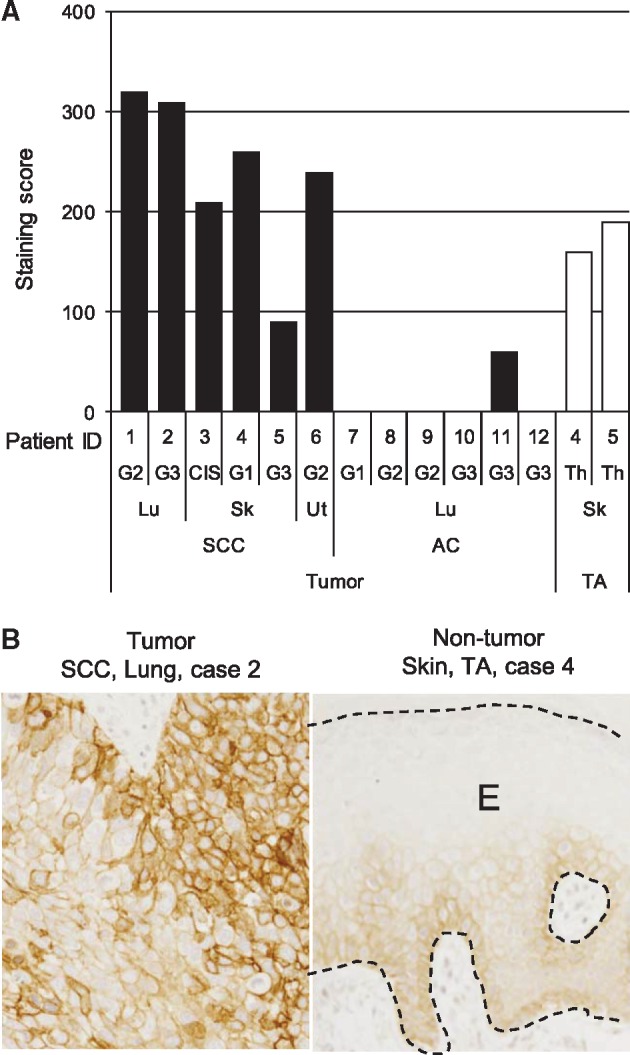
**Expression of DSG3 in human tissues.** (**A**) DSG3 was expressed frequently in SCC of various organs compared with adenocarcinoma of the lung. Tumour-adjacent skin also expressed DSG3. G1–G3, histology grade; CIS, carcinoma *in situ*; Th, thickening of epidermis; SCC, squamous cell carcinoma; AC, adenocarcinoma; TA, tumour-adjacent tissue; Lu, lung; Sk, skin; Ut, uterus. (**B**) Images of IHC staining in SCC of the lung (left panel) and tumour-adjacent skin (right panel). Labelled streptavidin-biotin method. E presents epidermis. Bar = 100 µm.

Six out of six cases of SCC in various organs were positive for DSG3 (Fig. 1A). The staining of a representative sample is shown in Fig. 1B (left panel). In 5/6 cases, the staining score was higher than 200, and two of the lung SCC cases had scores higher than 300. In contrast, only 1/6 cases was positive for adenocarcinoma in the lung, and the score was notably lower than lung SCC. In non-cancerous tissue, the epidermis in tumour adjacent skin was positive in 2/2 cases (Fig. 1A). DSG3 expression was observed from the basal cell layer to prickle cell layer (Fig. 1B, right panel). Epidermal hyperplasia was present, but the staining score was lower than 200 and tended to be lower compared with SCC.

### Generation of anti-mouse DSG3 mAbs with ADCC activity

**Fig. 2:**
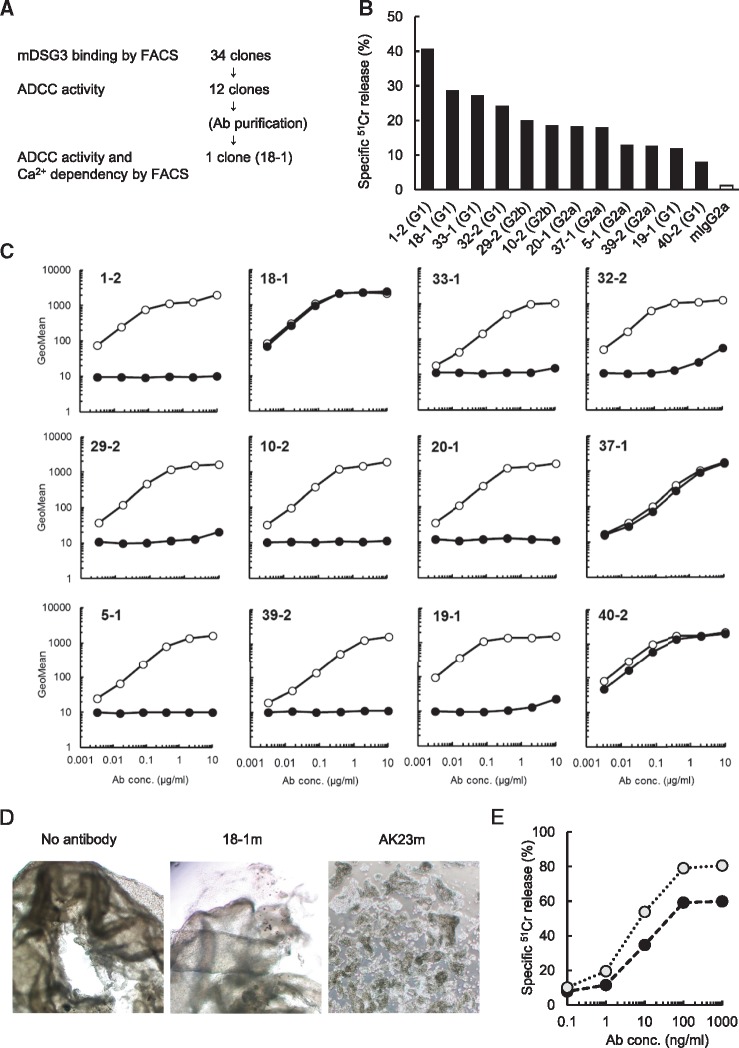
**Characterization of anti-mouse DSG3 mAbs**. (**A**) Screening flow for an anti-mouse DSG3 mAb. (**B**) Analysis of ADCC activity. ^51^Cr-labelled 1 × 10^4^ mouse DSG3/BaF3 cells were incubated with hybridoma supernatant at 1 μg/ml antibody for 15 min. mFcγRIIIa-NK92 were added at E/T ratio = 5:1 and the cell suspensions were further incubated at 37°C for 4 h. The radioactivity was measured with Gamma counter. Specific ^51^Cr release was calculated. Mouse IgG2a was used as reference control. (**C**) Analysis of Ca^2+^-dependent binding activity to mouse DSG3. Serial diluted mAbs were incubated with mouse DSG3/DG44. The binding activity to mouse DSG3 was measured with FACS with EDTA (closed circle) or without EDTA (open circle). (**D**) Analysis of cell–cell dissociating activity with mouse IgG2a type chimeric anti-DSG3 18-1m. Cell–cell dissociating activity with mAb 18-1m was analysed by keratinocyte dissociation assay (27). mAb AK23m was used as positive control. (**E**) ADCC enhancement by defucosylated chimeric anti-DSG3. ADCC activity of 18-1m was compared. Mouse DSG3/DG44 was incubated with serial diluted antibody and mixed with mFcγRIIIa-NK92 as effector cells. ADCC activity was measured. Black circle: 18-1m, grey circle: df-18-1m prepared with GFT (-/-) CHO cells.

To confirm the proof of concept in non-clinical models, we attempted to generate an anti-mouse DSG3 mAb that has anti-tumour activity by ADCC to SCC, but with no cell–cell dissociating effects in keratinocytes that cause PV (Fig. 2A).

By FACS with hybridoma supernatants, 34 clones with robust binding activity were selected. Next, ADCC activity was measured with an antibody concentration of 1 μg/ml, and 12 clones with the strongest ADCC activity were further selected (Fig. 2B).

### Selection of clones with no PV-like effects by evaluation of Ca^2^^+^-dependent binding and the cell–cell dissociation assay

The above-mentioned 12 clones were examined for Ca^2+^-dependent binding to mouse DSG3 by FACS. As it is well-known that pathogenic anti-DSG3 antibodies like AK23 recognize an epitope with a calcium-dependent conformation, we screened the antibodies for calcium dependency (28, 33). Three clones, 18-1, 37-1 and 40-2, bound to mouse DSG3 equally with and without Ca^2+^, and were determined as having Ca^2^^+^ independent binding activity (Fig. 2C). Other clones bound weakly to mouse DSG3 when Ca^2+^ was not present, and were determined as having Ca^2+^-dependent binding activity.

Of the three clones with Ca^2^^+^ independent binding activity, clone 18-1 had the highest affinity, and so was selected for further evaluation. In order to ascertain the potential to induce PV, cell–cell dissociating activity in a mouse keratinocyte sheet was tested. The test was designed to compare the cellular effects induced by a difference only in antigen binding while the Fc function remained the same.

18-1 and AK23 were genetically engineered into mouse IgG2a-type chimeric antibodies 18-1m and AK23m, and were subjected to the assay. 18-1m did not induce keratinocyte dissociation, while the positive control antibody AK23m did. This was thought to show that 18-1m was not pathogenic (Fig. 2D).

### Enhancement of ADCC activity by defucosylation of antibody

As reported previously, an antibody produced by GFT (-/-) CHO cells is mainly a fucose-free oligosaccharide (7), a so-called defucosylated antibody, which binds strongly to mouse FcγRIIIa and thus exhibits much enhanced ADCC. A defucosylated antibody, df-18-1m, induced ADCC activity that was comparable to a 10-fold concentration of 18-1m. Thus we confirmed that defucosylation further enhanced ADCC (Fig. 2E).

### Anti-tumour efficacy and toxicity of 18-1m, df-18-1m antibodies in vivo

As we were able to generate an anti-mouse DSG3 antibody with the potential of anti-tumour ADCC activity but no pathogenic activity to normal tissues, we examined the proof of concept in a syngeneic mouse model of a mouse lung SCC cell line, LC12.

**Fig. 3:**
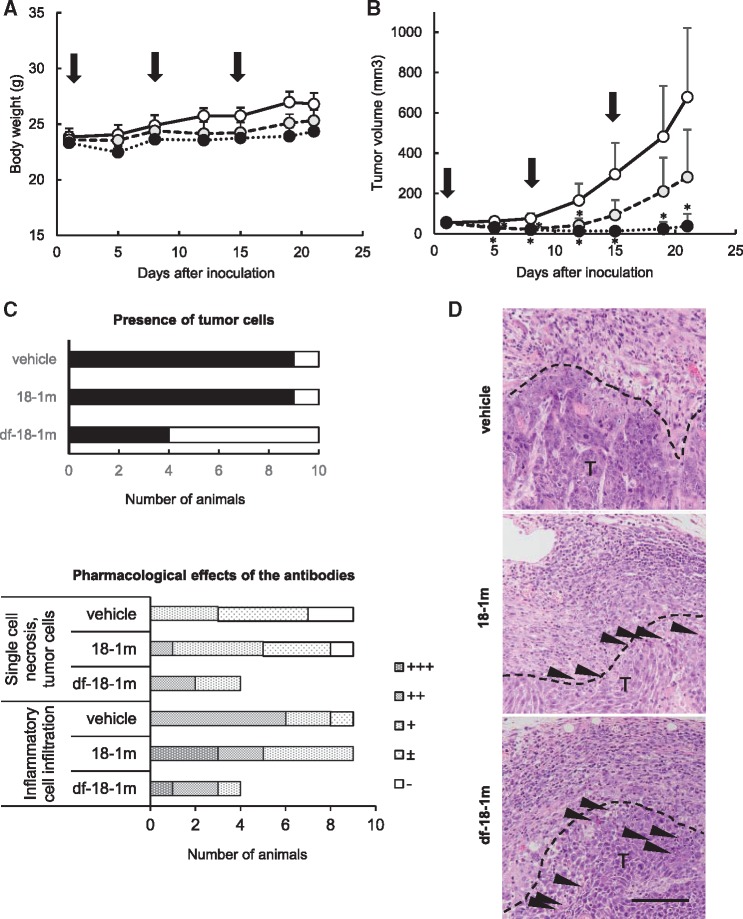
**Results of anti-mouse DSG3 mAb administration in tumour-bearing Balb/c mice**. Anti-mouse DSG3 mAb 18-1m and df-18-1m were inoculated at 10 mg/kg in LC12 syngeneic mouse model. Days of treatment with mAb are shown by arrows. (**A**) Body weight change. (**B**) Tumour volume change. The mean for each group is shown for each time point. Bars indicate standard deviation of the mean. White circle, control; grey circle, 18-1m; black circle, df-18-1m. *P < 0.05, compared with vehicle. (**C**) Histological examination of engrafted LC12 tumours. The presence of tumour cells in the site of engraftment (upper left). Closed square, present; open square, absent. Changes related to the pharmacological effects of the antibodies (lower left) are shown by severity of change: ±, very slight; +, slight; ++, moderate; +++, severe. (**D**) Representative images of tumours in each group. Increased inflammatory cell infiltration and single-cell necrosis (arrow heads) were observed in antibody administered tumours. The changes were noted around the interface (broken line) of the tumour mass (T) with surrounding tissue. Bar = 100 µm.

After administering 18-1m or df-18-1m once a week for 3 weeks to LC12-inoculated mice, the body weight of antibody-administered mice was slightly lower compared with that of vehicle-administered mice at the end of the study. However, there were no changes in the general condition of mice in any of the groups and there is no serious toxicity (Fig. 3A).

In contrast, the tumour volume was significantly lower antibody-administered groups compared with the control group (Fig. 3B). Suppression of tumour growth was marked in the df-18-1 group compared with the 18-1m group. The number of cases with tumour volumes of less than 3 mm^3^ or with no palpable mass of histologically detectable tumour cells was 1/10 in each of the control and 18-1m groups, but 6/10 in the df-18-1m group (Fig. 3C). Histopathologically, single-cell necrosis of tumour cells and inflammatory cell infiltration were observed in palpable tumours of the 18-1m group. Similar changes were observed in the palpable tumours of the df-18-1m group (Fig. 3C and D).

There were no histopathological changes in tissues containing squamous cells (skin, oral mucosa, oesophagus and forestomach) and other tissues (intestine, colon, liver, kidney, spleen and thymus).

### Generation of anti-human DSG3 antibody

The results from the mouse studies showed the possibility that antibodies with anti-tumour activity and no PV-like pathogenic activity could be generated, so we proceeded to generate an anti-human DSG3 antibody with similar features.

**Fig. 4:**
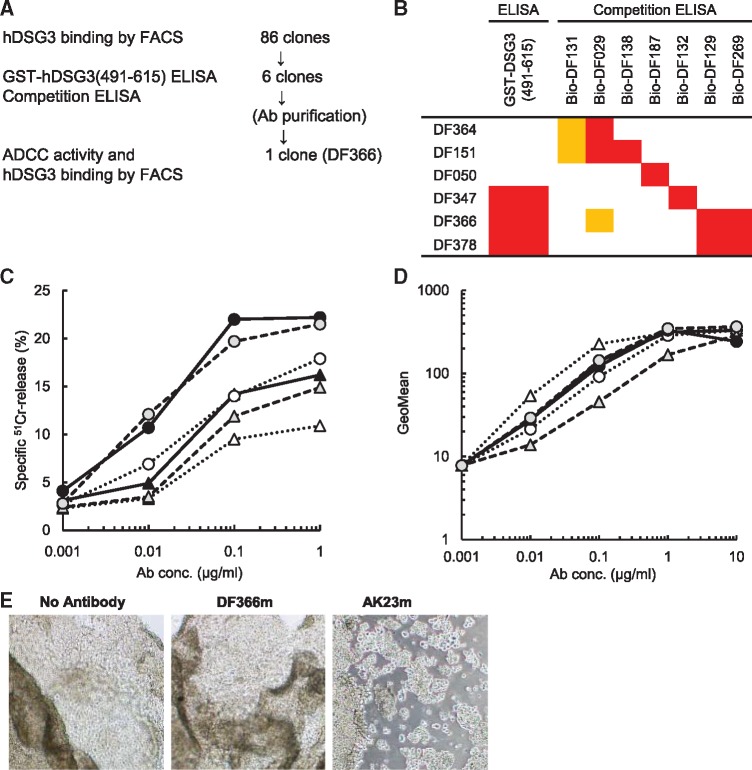
**Characterization of anti-human DSG3 mAbs.** (**A**) Screening flow for an anti-human DSG3 mAb. (**B**) Classification of mAbs with GST-human DSG3 (aa 491-615) ELISA and competition ELISA. In GST-ELISA, dark grey indicates positive. In competition ELISA, dark grey indicates >90% inhibition and light grey indicates >70% inhibition. Bio-(antibody number) indicates a biotinylated antibody. (**C**) ADCC activity was measured as specific ^51^Cr release. Black circle, DF366c; grey circle, DF378c; white circle, DF347c; black triangle, DF151c; grey triangle, DF364c; white triangle, DF050c. (**D**) Binding activity to human DSG3/DG44. Serial diluted antibody was incubated with human DSG3/DG44, and binding activity was measured with FACS. Symbols are the same as (C). (**E**) Cell–cell dissociation activity was analysed in human keratinocytes. Mouse IgG2a chimeric antibody DF366m was compared with AK23m as a positive control.

The screening flow in Fig. 4A shows how the antibody was selected. We immunized mice with human sDSG3-mIgG2aFc protein, and established 86 hybridoma clones that were positive for activity against human DSG3, from which clones with high affinity were selected by FACS. In general, the epitopes that are contained in the membrane-proximal region of a membrane protein tend to give high ADCC (26, 31), so hDSG3 (aa 491-615, which is equivalent to the membrane-proximal region) would be a candidate for an ADCC epitope. Therefore, we added a GST-DSG3 ELISA to the screening to obtain an antibody that would bind to a region that is safely distant from the EC1-EC2 domains and would have higher ADCC.

To select clones that bound to a unique binding region, we used a competition ELISA in the following procedure. First, seven of the hybridoma clones that gave no cross-reaction were chosen as competition antibodies and were biotinylated. Then, the competition ELISA divided the hybridoma clones into six groups, and a representative clone from each group was selected after evaluating the binding affinity by FACS and the ADCC. Chimeras of each of these clones were generated with a human IgG1 Fc portion to compare the cytotoxic effects in a clinically relevant format.

Figure 4B summarizes the characters of the six representative clones. Their ADCC was categorized into two levels of activity: high (two clones) and middle (four clones) (Fig. 4C). As expected, the binding regions of the two clones with the strongest ADCC, DF366 and DF378, were located between aa 491 and 615. There was no difference in binding affinity of the two clones with high ADCC (Fig. 4D).

From these results, DF366 was selected as the clone with strongest ADCC activity and converted to a mouse IgG2a subclass (designated as DF366m) to evaluate cell–cell dissociation activity in human keratinocyte sheets. DF366m did not show cell–cell dissociation activity, while the positive control AK23m antibody did (Fig. 4E).

### Anti-tumour efficacy of anti-human DSG3 mAbs against SCC

**Fig. 5:**
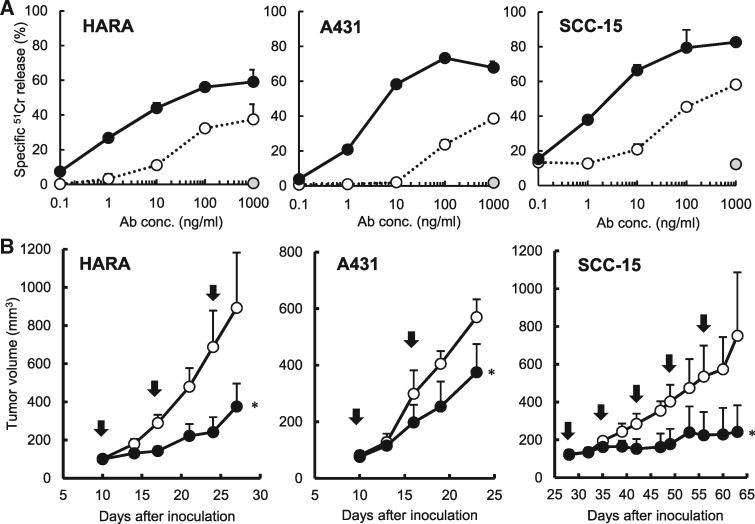
**Anti-tumour activity of defucosylated anti-human DSG3.** (**A**) ADCC activity was measured with SCC cell lines. Bars indicate standard deviation of the mean. HARA, human lung SCC; A431, human skin SCC; SCC-15, human tongue SCC. DF366m (white circle), df-DF366m (black circle), control mIg2a (grey circle). (**B**) Each SCC cell line was inoculated s.c. in SCID mice. Mice were treated with mAb df-DF366m (black circle) or vehicle, PBS (white circle) on the days indicated by arrows. Tumour volume was measured twice a week. Bars indicate standard deviation of the mean. *P < 0.05, compared with vehicle.

ADCC activity of DF366m was compared with a defucosylated antibody (df-DF366m) with mFcγRIIIa-NK92 as the effector cells. The anti-tumour effect of ADCC-enhanced df-DF366m was evaluated in an *in vitro* ADCC assay with human SCC cell lines HARA, A431 or SCC-15. The results showed that both DF366 and df-DF366m have ADCC activity against human SCC cell lines (Fig. 5A). df-DF366m showed strong ADCC activity even at concentrations that were several score times lower than DF366m.

Next, the anti-tumour effect of df-DF366m was investigated *in vivo* using xenograft models of HARA, A431 and SCC-15. In all models, efficacy of df-DF366m was significantly enhanced (Fig. 5B). Especially in SCC-15, the tumour growth was completely inhibited.

## Discussion

DSG3 has been reported to be highly expressed in lung SCC both at mRNA and protein levels (16, 17, 29). We examined the expression of DSG3 in SCC by IHC and confirmed that the expression was high, which was consistent with the reported results, and thus we considered that DSG3 is a potential target for therapeutic antibodies against SCC. On the other hand, DSG3 is also expressed in normal squamous epithelium (16), and autoantibodies against DSG3 cause PV, an autoimmune disease that includes severe skin lesions, such as blistering (30). Tsunoda *et al.* have also shown that anti-DSG3 mAbs induced PV blisters in a passive transfer assay with neonatal mouse (15). Because of those results, we judged it necessary to avoid PV-like pathogenic activity if we selected DSG3 as a target for a therapeutic antibody. We generated anti-DSG3 mAbs to examine their potential as a therapeutic antibody.

It is well-known that therapeutic antibodies can induce biological effects by several mechanisms. To attack cancer cells, a variety of modes of action have been utilized: mAb with ADCC, CDC activity that utilizes the host immune response, toxin-conjugated antibodies that utilize antibodies as a drug delivery system and so on (6, 10). The ADCC activity of an antibody is considered to be dependent on the amount of antigen available to induce host immune response and cytotoxicity (7, 31, 32). In this study, we have found that some SCC cases express higher levels of DSG3 than non-tumour. Thus we hypothesized that a mode of action via ADCC would be effective for obtaining tumour specificity.

In order to achieve our concept, it was thought necessary to generate an antibody with no PV-like pathogenic activity. It has been reported that in the pemphigus pathogenesis the neutralizing activity of patient pathogenic autoantibodies causes a disruption of the DSG3-DSG3 adhesive interaction, and that subsequently destroys the epithelial cell structure (15). It is also known that autoantibodies with a Ca^2+^-sensitive epitope cause cell–cell dissociation, and that these antibodies recognize a Ca^2+^-dependent conformation epitope that regulates cell–cell adhesion function (33). As epitope specificity is critical for PV activity, we considered it possible to add the ADCC function to an antibody while avoiding PV activity by selecting an appropriate epitope. Thus we attempted to obtain a wide variety of antibodies for functional screening.

In order to generate a wide variety of antibodies we utilized KO mice for immunization as described previously (34, 35). Additionally we applied a DNA immunization method and were able to obtain a large number of different antibodies. Next, we adopted functional screening methods to select antibodies that have ADCC activity, but do not cause PV-like action. Conventional assay systems with mouse splenocytes are known to yield inconsistent results between lots, and the preparation is also a complicated process. Thus we established an ADCC screening system with the mFcγRIIIa-NK92 cell line that could stably measure ADCC activity. To screen for mAbs with no PV-like effects, we tested binding to DSG3 in a Ca^2^^+^ independent manner by ELISA and FACS in the presence of a Ca^2+^ chelating agent, EDTA. As a result we obtained anti-mouse DSG3 antibodies with ADCC activity *in vitro*, but no keratinocyte cell–cell dissociation.

We investigated the binding region of these clones by domain-swapped mutant analysis, and found that the mouse clone 18-1 binds to a region in the cadherin domains not EC1, while DF366 recognizes the membrane-proximal region (data not shown). When screening for anti-human DSG3 antibodies, we could not obtain antibodies that were cross-reactive to mouse DSG3. Mouse and human DSG3 are very homologous apart from their membrane-proximal regions, which tend to contain binding regions that provide high ADCC. When we screened for the anti-human DSG3 antibody, we aimed for an antibody that bound to a region that induced higher ADCC and was safely distant from the EC1-EC2 domains. With this in mind, we considered the best antibody against human DSG3 would be derived from a clone that bound to the proximal region. We therefore used a competition ELISA to classify the clones according to their binding regions and then selected the antibody in each group with the highest ADCC.

As ADCC activity and PV-like activity are different modes of action, we considered that enhancing ADCC would be an effective approach to improve anti-tumour activity without causing a toxic effect to the skin. To this end, we chose the mouse antibody that had the highest ADCC activity and enhanced its ADCC activity by changing to an IgG2a version (36, 37) and a defucosylated version of the antibody using the GFT (-/-) CHO cells (7). Then, to test our hypothesis *in vivo*, we evaluated the non-defucosylated and defucosylated antibodies for anti-tumour activity and PV-like pathogenic activity in a mouse model. *In vivo* anti-tumour activity was observed with both antibodies, but the enhanced ADCC activity did not lead to additional weight loss or induction of severe toxicity, such as patchy hair loss, oral erosion and death, which are reported in mice dosed with PV-inducible anti-DSG3 AK23 (38). In a preliminary study with another mouse model, we observed minor, non-lethal changes in the vaginal mucosa, and we are currently studying the lesions in detail. From these results we judged that the defucosylated antibody improved anti-tumour activity, while the enhanced ADCC activity was not associated with enhanced toxicity. The current results in the mouse model strongly suggest that the neutralizing activity that disrupts DSG3 adhesive interaction can be separated from cytotoxicity by ADCC to DSG3-expressing cells. Therefore DSG3 was thought to be a suitable target for therapeutic antibody development.

Because the potential of DSG3 as a therapeutic target was shown in a mouse model, we attempted to generate an anti-human DSG3 antibody with similar characteristics to the mouse antibody. We succeeded in generating an antibody that binds to DSG3, with ADCC activity *in vitro*, and no adhesive interference activity to keratinocytes. Defucosylated anti-human DSG3 antibody showed anti-tumour activity against three differential SCC cell lines in xenograft models. These results indicate that it is possible to generate an anti-human DSG3 antibody with the same properties as our anti-mouse DSG3 antibody. It should be noted that effects in normal tissues could not be evaluated in mouse xenograft models with anti-human DSG3 antibody because the antibody does not recognize mouse DSG3. To address these issues, future toxicity studies will be necessary.

Ideally, therapeutic antibodies that function by cytotoxicity to cancer cells should target an antigen that is tumour-specific with no expression in normal tissues. However such tumour-specific antigens are very rare (39). Here we show it is possible to develop novel anti-tumour therapeutic antibodies even if the target molecule is expressed in both tumour and normal tissues, by carefully selecting the appropriate epitope and mode of action. Such an approach may enable currently undruggable targets to be targeted in the future.

## Supplementary Material

Supplementary Table

## Abbreviations

ADCC: antibody-dependent cell cytotoxicity
DSG3: desmoglein 3
FACS: flow cytometry
mAb: monoclonal antibody
PV: pemphigus vulgaris
SCC: squamous cell carcinoma
