# Supplementary material for: Generation of an anti-desmoglein 3 antibody without pathogenic activity of pemphigus vulgaris for therapeutic application to squamous cell carcinoma
Source: J Biochem. 2018 Sep 18;164(6):471–81. doi: 10.1093/jb/mvy074 (PMC6267343; doi:10.1093/jb/mvy074)
Supplement: Supplementary Table [file mvy074_supplementary_table.docx]

## **Supplementary Table**

| Cell line | Culture medium |
| --- | --- |
| CHO DG44 | CHO-S-SFM II with hypoxanthine and thymidine |
| Ba/F3 | RPMI-1640, 10% FBS, 1 ng/ml mouse IL-3 (R&D systems) |
| HARA | RPMI-1640, 10% FBS |
| A431 | DMEM (high glucose), 10% FBS |
| SCC-15 | 1:1 mixture of DMEM and Ham F-12, 1.5 g/l sodium bicarbonate, 2.5 mM L-glutamine, 400 ng/ml hydrocortisone (Sigma), 10% FBS |
| NK-92 | Alpha minimum essential medium without ribonucleosides and deoxyribonucleosides with L-glutamine, penicillin, streptomycin, 0.2 mM inositol (Sigma), 0.1 mM 2-mercaptoethanol, 0.02 mM folic acid (Sigma), 100 U/ml recombinant human interleukin-2 (Peprotech), 12.5% horse serum, 12.5% FBS |
| P3U1 | RPMI-1640, 10% FBS |
| CryoNHEK-Neo | KGM-Gold™ Keratinocyte Growth Medium BulletKit™ （Lonza） |
| MPK-BL6 | CnT-PR medium (CELLnTEC) |
